# Challenges of Reintegration and Recidivism Among Older Adult Prisoners in Ghana

**DOI:** 10.1093/geroni/igaf122.2666

**Published:** 2025-12-31

**Authors:** Mathias Adjei, Eric Frimpong, Akwasi Adjei Gyimah, Spendilove Yankson

**Affiliations:** Miami University, Oxford, Ohio, United States; University of Massachusetts Boston, Boston, Massachusetts, United States; Miami University Scripps Gerontology Center, Oxford, Ohio, United States; University of Cape Coast, Cape Coast, Central, Ghana

## Abstract

As the global aging prison population rises, understanding the challenges of reintegration for older adult prisoners is critical in developing age-responsive correctional policies. This study uses a Life Course Perspective to explore factors contributing to reintegration and recidivism among older adult prisoners in Ghana. Using a qualitative approach, data were gathered from 21 participants. This included 8 older inmates aged 60 + (with at least two prior incarcerations) and 5 prison officers from the Sunyani and Kumasi central prisons. In addition are 8 community members. Findings highlight that the lack of age-appropriate rehabilitation programs for older inmates presents a major barrier to successful reintegration. Older adult prisoners face challenges in accessing the necessary support and resources to address age-related needs such as healthcare, skill-building, and emotional well-being. Additionally, societal stigma, where former prisoners are still considered criminals, hinders their acceptance by family, communities, and potential employers. The persistence of these negative perceptions reinforces discrimination in social relationships and employment. This led some individuals to engage in criminal activities, with the intent of returning to an environment where they feel belonged. Participants further indicated that they lack the necessary resources essential to provide age-specific rehabilitation programs creating a barrier to their successful reentry into society. Policy reforms should focus on age-responsive reintegration programs, addressing healthcare, vocational training, and social support while tackling societal stigma. Adequate resources and collaboration are key to reducing recidivism and ensuring successful reentry into society.

